# Hierarchical 3D FeCoNi Alloy/CNT @ Carbon Nanofiber Sponges as High-Performance Microwave Absorbers with Infrared Camouflage

**DOI:** 10.3390/ma18010113

**Published:** 2024-12-30

**Authors:** Yifan Fei, Junya Yao, Wei Cheng, Wenling Jiao

**Affiliations:** Shanghai Frontiers Science Research Center of Advanced Textiles, Engineering Research Center of Technical Textiles (Ministry of Education), Key Laboratory of Textile Science & Technology (Ministry of Education), College of Textiles, Donghua University, Shanghai 201620, China

**Keywords:** high-elastic sponges, CNT, infrared camouflage, microwave absorption, RCS simulation

## Abstract

Microwave absorbers with infrared camouflage are highly desirable in military fields. Self-supporting 3D architectures with tailorable shapes, composed of FeCoNi alloy/carbon nanotubes (CNTs) @ carbon nanofibers (CNFs), were fabricated in this study. On the one hand, multiple loss mechanisms were introduced into the high-elastic sponges. Controllable space conductive networks caused by the in situ growth of CNTs on the CNFs contributed to the effective dielectric and resistance loss. Moreover, the uniformly distributed magnetic alloy nanoparticles (NPs) with dense magnetic coupling resulted in magnetic loss. On the other hand, heterogeneous interfaces were constructed by multicomponent engineering, causing interfacial polarization and polarization loss. Furthermore, the internal structures of sponges were optimized by regulating the alloy NPs sizes and the growth state of CNTs, then tuning the impedance matching and microwave absorption. Therefore, the high-elastic sponges with ultra-low density (7.6 mg·cm^−3^) were found to have excellent radar and infrared-compatible stealth properties, displaying a minimum refection loss (RL_min_) of −50.5 dB and a maximum effective absorption bandwidth (EAB_max_) of 5.36 GHz. Moreover, the radar stealth effect of the sponges was evaluated by radar cross-section (RCS) simulation, revealing that the multifunctional sponges have a promising prospect in military applications.

## 1. Introduction

The widespread implementation of communication technologies, such as 5G, IoT, and WLANs, has profoundly influenced human life. These technologies have become essential components of contemporary society, facilitating various devices, including communication equipment, household appliances, and aerospace systems, enhancing our quality of life. Nevertheless, the rapid proliferation of electronic devices has led to increased electromagnetic radiation, which may disrupt the normal operation of these devices and pose potential risks to human health. Microwave absorbers (MAs) can dissipate electromagnetic wave energy by converting it into heat. The MA is currently utilized across various sectors, including the military, aviation, communications, electronics, and medicine [1,2]. However, in practical applications, the energy conversion characteristic of MAs makes the target vulnerable to detection by infrared sensors. Thus, high-performance microwave absorbers with infrared camouflage have become a research hotspot [3,4,5,6]. In recent years, carbon-based three-dimensional (3D) architectures (e.g., sponges [7,8], aerogels [9], and mesh [10,11]), compounded from one-dimensional (1D) carbon nanotubes (CNTs) or carbon nanofibers (CNFs), have attracted attention continuously. The carbon 3D interconnection network possesses prominent internal pore structures, which can induce multiple electromagnetic wave scattering [12]. According to the Maxwell–Garnett theory, an increase in the pore volume will lead to a reduction in the effective dielectric constant, thereby contributing to an improvement in impedance-matching properties [13,14,15]. Currently, multicomponent recombination (e.g., magnetic substances [16], dielectric materials [17,18]) and heterogeneous interface engineering [19] are regarded as crucial approaches to improving microwave absorption properties. Component contact recombination can form a heterogeneous interface inevitably, which not only inherits the intrinsic properties of individual components but also adjusts the alignment of energy bands at the heterogeneous interface region, forms crystal defects, and accumulates space charges at the phase boundary [20]. For instance, Liu et al. [21] prepared a magnetic neodymium oxide @ CNF aerogel with a minimum RL of −63.7 dB at a thickness of 3.17 mm and an EAB_max_ of 7.44 GHz. The introduction of neodymium oxide enhances the magnetic loss of the carbon-based 3D network, relying on the magnetic coupling between magnetic NPs and magnetic–dielectric balance to optimize the impedance matching. Du et al. [22] reported silicon carbide nanowires coated on CNF aerogels, changing the interfacial electron distribution to construct built-in electromagnetic fields and spatial electric dipole moments, of which the RL_min_ is −53.3 dB at 17.7 GHz. Most currently available high-performance carbon-based 3D interconnection network MAs exhibit a deficiency in skeleton strength, which ultimately restricts their utility in practical applications. The introduction of one-dimensional carbon nanostructures represents an effective solution. The 1D carbon nanostructure enhances the skeleton strength and lowers the percolation threshold of the 3D network [23]. Owing to the quantum size effect, many defect sites exist in the 1D carbon nanostructure, providing polarization centers to realize polarization loss [23]. For instance, Wu et al. mentioned that the MXene/CNF mesh structure exhibited superior microwave absorption (MA) performance, resulting in a minimum refection loss (RL_min_) of −53.02 dB and a maximum effective absorption bandwidth (EAB_max_) of 5.3 GHz [24]. Wang et al. reported iron sulfide-doped honeycomb porous CNF aerogels, showing a substantial absorption value of −37 dB at 15.7 GHz and an EAB_max_ of 5.52 GHz at a thickness of 2 mm [25].

Infrared stealth materials mainly include low-emissivity materials (e.g., metal [26], carbon [27]), thermal insulation materials [28,29], and phase-change materials [30]. Metal or carbon-based materials absorb and reflect infrared to achieve an infrared stealth effect. Notably, CNTs have MA and infrared stealth double characteristics [28]. For instance, Wang et al. [31] reported that CNT/Zn_0.96_Co_0.04_O complex fibers possessed radar and infrared-compatible stealth properties. When the mass fraction of CNTs was 6%, the infrared emittance was as low as 0.61. Concurrently, the microwave was converted into thermal energy dissipation, attributed to the resistance and dielectric loss due to the dielectric relaxation and surface polarization in CNTs [32]. Self-supporting 3D interconnection networks often have superior thermal insulation properties due to the abundant porous structures [33]. Thermal insulation coating can control the surface temperature of the target object, reducing the intensity of infrared radiation to realize infrared stealth. Thus, carbon-based 3D architectures are expected to achieve dual radar/infrared stealth.

Herein, we report FeCoNi alloy/carbon nanotubes @carbon nanofiber (FCCF) sponges with 3D porous crosslinked structures via a simple conjugated electrostatic spinning technique and subsequent heat treatments. Firstly, precursor sponges with a 3D interconnected fiber network were prepared by conjugated electrostatic spinning. Subsequently, the precursor sponges were carbonized to form carbon-based self-supporting 3D architectures, composed of CNFs with magnetic metal NPs distributed evenly. The interior of the FCCF sponge is composed of a multitude of CNFs and CNTs, which enhance the 3D skeleton strength. Additionally, the sponge exhibits notable resilience and fatigue resistance. Subsequently, the dimension and arrangement of the metal nanoparticles (Fe, Co, and Ni alloys) were modified by altering the concentration of the metal compounds present in the spinning solution. The process enabled the manipulation of the in situ growth of CNTs, magnetically coupled networks, and heterogeneous interface engineering. As a result, the FCCF sponges exhibit excellent MA and infrared stealth-compatible properties, aligning with the current trend of multifunctional development in MA. Our work promotes the development of flexible microwave-absorbing materials combined with infrared stealth function.

## 2. Experimental Section

### 2.1. Materials

Polyacrylonitrile (PAN, M_w_ = 150,000) was supplied by Macklin Biochemical Co., Ltd., (Shanghai, China). Iron (III) acetylacetonate (98%) was purchased from Shanghai Aladdin Biochemical Technology Co., Ltd. (Shanghai, China) Cobalt (III) acetylacetonate (98%) was supplied from SIGMA·ALDRICH, Co., Ltd. (St. Louis, MO, USA) Nickel (II) chloride hexahydrate (NiCl_2_·6H_2_O, AR) and N, N-dimethylformamide (DMF, 99%) were obtained from Sinopharm Chemical Reagent Co., Ltd. (Shanghai, China).

### 2.2. Preparation of Electrospinning Solution

PAN was slowly added to DMF under magnetic stirring so that it was completely dissolved in DMF. Then, iron acetylacetonate, cobalt acetylacetonate, and nickel chloride hexahydrate were added sequentially. The total amount of metal compounds was 1.05 g, and the molar ratio of the three components was 5:4:1. Finally, after magnetic stirring for six hours, the spinning solution was obtained.

### 2.3. Preparation of FCCF and FCF Sponges

At the beginning of the two syringes pointing simultaneously to the center of the rotor, both sprayed filaments on the surface of the rotor lie flat and stacked, forming a more compact structure of the underlying structure. Then, by adjusting the angle of the two syringes and applying an additional voltage, the filaments from both syringes are entangled with each other on the outside of the rotor, forming a loose fiber aggregate similar to a spider web, which is then coiled and collected by the rotor to form a fluffy sponge structure. Both syringes were charged with a voltage of approximately 10 KV during the preparation process. The liquid was supplied at a rate of 0.8 mL/h, with a receiving distance of 12 cm and an angle of 30° between the syringe and the center line of the rotor. The ambient humidity and temperature were 55% and 23 °C, respectively.

Subsequently, the prepared precursor was dried at 60 °C for 12 h, then heat-treated in the air at 250 °C for 2 h with a ramp rate of 3 °C/min, and then carbonized at 700 °C for 2 h with a ramp rate of 2 °C/min in an argon atmosphere. Finally, the sample sponge was obtained. Under the same molar ratio of iron, cobalt, and nickel of total metal salts, the mass ratios of total metal and PAN were adjusted to 0.8:1, 1:1, and 1.2:1, and the corresponding sponges were marked as FeCoNi alloy/CNT @ CNF-1 (FCCF-1), FCCF-2, and FeCoNi alloy @ CNF (FCF), respectively.

### 2.4. Material Characterization

Scanning electron microscopy (SEM, Hitachi Regulus 8100, Tokyo, Japan) and transmission electron microscopy (TEM) were used to characterize the microstructure of the sponges. X-ray diffraction (XRD, Bruker D8 ADVANCE, Berlin, Germany) was used to characterize the phase constitution of the sponge. Raman spectroscopy (Raman, Renishaw inVia-Reflex, London, UK) was used to characterize the degree of graphitization of the sponge. Analysis of the chemical state of elements on the sponge surface was conducted using X-ray photoelectron spectroscopy (XPS, Thermo Escalab 250Xi, Waltham, MA, USA). The compression recovery of sponges was recorded with a dynamic mechanical analyzer (DMA, TA DMA850, New castle, DE, USA). Temperature changes in the sponge were captured using a thermal infrared imager (Fluke TiS75+, Everett, WA, USA). The electromagnetic parameters of complex permittivity and complex permeability variations were measured by the coaxial line method using a vector network analyzer (VNA, Agilent PNA-L N5234B, Santa Clara, CA, USA) during the frequency range of 2 to 18 GHz. In this test, 30 wt% of the sample was mixed well with 70 wt% of molten paraffin, injected into a mold, and pressed to form a coaxial ring with an outer diameter of 7 mm and an inner diameter of 3.04 mm.

In order to obtain better experimental results, the paraffin filling ratio of the FCF samples was adjusted to 75 wt%. According to transmission line theory, the reflection loss (RL) can be calculated from the following equation [34,35]:

(1) $$Z_{in}=Z_{0}{\left(\mu_{r}/\varepsilon_{r}\right)}^{1/2}\tanh\left[j\left(2\pi fd/c\right){\left(\mu_{r}\varepsilon_{r}\right)}^{1/2}\right]$$

(2) $$RL=20\log\left|{(Z}_{in}-Z_{0})/\left(Z_{in}+Z_{0}\right)\right|$$

where *Z_in_* is the input impedance, *Z*_0_ is the impedance of air, *f* is the frequency, *t* is the thickness of the absorber, and $\mu_{r}$ and $\varepsilon_{r}$ are the complex permeability and complex permittivity of the absorber, respectively.

According to Debye’s relaxation theory, the real part and imaginary part of the complex permittivity can be expressed by the equation below [36]:

(3) $$\varepsilon^{\prime }=\varepsilon_{\infty }+\frac{\varepsilon_{s}-\varepsilon_{\infty }}{1+{\left(2\pi f\right)}^{2}\tau^{2}}$$

(4) $$\varepsilon^{\prime \prime }=\frac{2\pi f\tau \left(\varepsilon_{s}-\varepsilon_{\infty }\right)}{1+{\left(2\pi f\right)}^{2}\tau^{2}}$$

(5) $${\left(\varepsilon^{\prime }-\frac{\varepsilon_{s}+\varepsilon_{\infty }}{2}\right)}^{2}+{\left(\varepsilon^{\prime \prime }\right)}^{2}={\left(\frac{\varepsilon_{s}-\varepsilon_{\infty }}{2}\right)}^{2}$$

where $\varepsilon^{\prime }$ is the real part of permittivity, $\varepsilon^{\prime \prime }$ is the imaginary part of permittivity, $\varepsilon_{\infty }$ represents the optical permittivity, $\varepsilon_{s}$ denotes the static permittivity, *f* is the frequency, and τ is the relaxation time.

The eddy current factor can be expressed by the equation below [34,37]:

(6) $$C_{0}=-\mu ”{\left(\mu \prime \right)}^{-2}f^{-1}=2\pi \mu_{0}d^{2}\sigma$$

where $\mu_{0}$ is the permeability in vacuum, *d* is the thickness of the absorbers, and *σ* is the electrical conductivity.

The attenuation constant *α* is a crucial parameter that describes a material’s ability to reduce EM waves. It can be calculated using the following equation [38]:

(7) $$\alpha =\frac{\sqrt{2}\pi f}{c}*\sqrt{\left(\mu^{”}\varepsilon^{”}-\mu^{\prime }\varepsilon^{\prime }\right)+\sqrt{{\left(\mu^{”}\varepsilon^{”}-\mu^{\prime }\varepsilon^{\prime }\right)}^{2}+{\left(\mu^{\prime }\varepsilon^{”}+\mu^{”}\varepsilon^{\prime }\right)}^{2}}}$$

### 2.5. RCS Simulation

To investigate the reaction of the sponge material to far-field electromagnetic waves, the radar cross section (RCS) was simulated using the CST studio suite 2022 software. Following a commonly used metal backplane model, the model is set up as a double-layered rectangle with a length and width of 20 × 20 cm. The bottom layer is an ideal electrically conductive layer (PEC) with a thickness of 2 mm, and the top layer is an absorber with a thickness of 2 mm. In detail, the sponge/PEC model is placed into the *X-O-Y* plane with linearly polarized electromagnetic waves incident in the positive direction from the *Z*-axis. Monitoring points are set at 0.2 GHz intervals from 2 to 18 GHz, with boundary conditions set to open (add space) in all directions. The RCS value can be derived from the following equation [39]:

(8) $$\sigma \left(dB\cdot m^{2}\right)=10log{\left[\left(4\pi S/\lambda^{2}\right)\left|E_{s}/E_{i}\right|\right]}^{2}$$

where *S* is the area of the model, *λ* represents the wavelength of the incident wave, and *E_s_* and *E_i_* are the intensities of the electric field of the scattered wave and of the incident wave.

The radar detection distance *λ* can be calculated by the following equation:

(9) $$dB\cdot m^{2}=10\times lg\left(m^{2}\right)$$

(10) $$RCS=k\frac{1}{\lambda^{4}}$$

## 3. Results and Discussion

### 3.1. Preparation and Characterization of the FCCF Sponges

**Figure 1:**
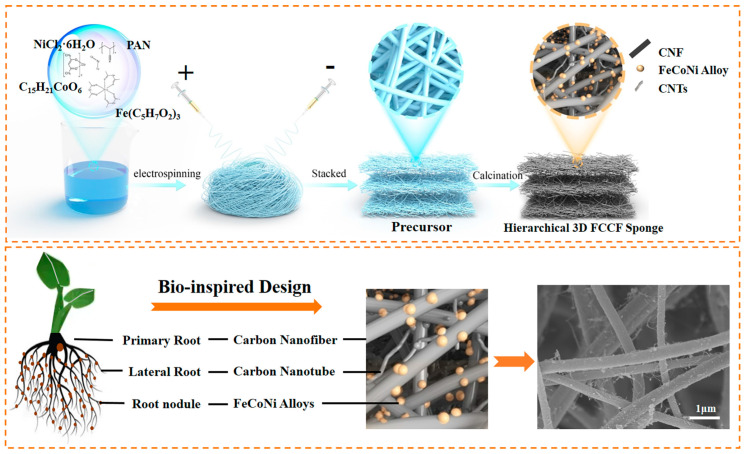
General procedure for the synthesis of layered FCCF sponges.

It is well established that the root system of plants is highly complex, comprising primary roots and lateral roots, which facilitate the fixation and extraction of nutrients by plants. Inspired by legumes’ root structures, we constructed a distinct hierarchical structure, where CNFs and CNTs are used to simulate the primary root and lateral roots, respectively. Similarly, the 3D conductive network would effectively capture electromagnetic waves. As demonstrated in Figure 1, FCCF sponges with a 3D porous cross-linked structure via simple conjugated electrostatic spinning technique and subsequent heat treatments were obtained. Specifically, heat treatment contains pre-oxidation and carbonization. In the pre-oxidation process, linear polyacrylonitrile molecules were converted into a conjugated trapezoidal structure, beneficial to obtaining dense and high-strength CNFs. Concurrently, FeCoNi metal compounds were oxidized, forming alloy oxides. Subsequently, the polyacrylonitrile nanofibers were completely carbonized into CNFs at 700 °C, accompanied by the formation of FeCoNi alloy with the CNFs used as a reductant. TGA images (Figure S1) showed that the mass loss of the precursor sponge was concentrated at 450–700 °C, indicating that the sponge component remained stable even if the calcination temperature was greater than 700 °C. Interestingly, CNTs were uniformly distributed on the CNFs, which constructed a distinctive 3D hierarchical conductive network. Notably, the size and spacing of FeCoNi alloy can affect the in situ growth process of CNTs on the CNF surface, which was tuned by changing the concentration of metal compounds, obtaining FCCF-1, FCCF-2, and FeCoNi alloy @CNF (FCF) sponges, respectively. Furthermore, the FCCF-2 sponge with a thickness of 3 cm exhibited an ultra-low density (7.6 mg/cm^3^), which can stand on the stamen, as shown in Figure S2 (Supporting Information).

The prepared FCCF sponge displays notable expandability in the field of microwave absorption. The material has a distinctive microstructure and exemplary microwave absorption characteristics, enabling superior attenuation of electromagnetic energy. Its attenuation properties can be tailored by manipulating the microstructure and chemical composition of the FCCF sponge, facilitating multi-band microwave absorption and meeting the requirements of diverse wavelength absorption scenarios. Secondly, due to its favorable mechanical properties and versatility in morphology, FCCF exhibits considerable potential for utilization in many fields, including flexible electronics, construction, and aerospace. From an economic standpoint, FCCF sponges prepared by electrostatic spinning are also viable. Electrostatic spinning is distinguished by its low cost and high production efficiency, with the requisite material cost also being relatively low, making it an economically feasible choice. However, the primary challenge for its large-scale preparation lies in the limitations of the electrostatic spinning scale, which also requires further investigation and resolution in the future.

**Figure 2:**
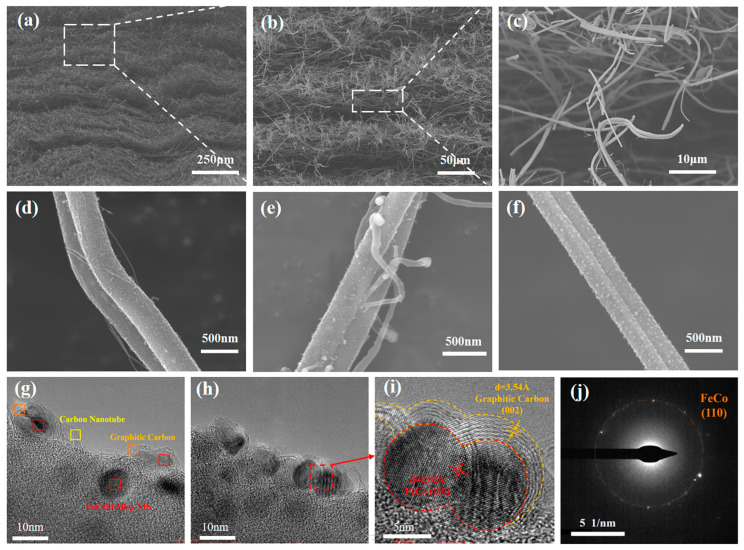
(**a**–**c**) SEM images of the FCCF-2 sponge with different magnifications. (**d**–**f**) SEM images of FCCF-1, FCCF-2, and FCF sponges. (**g**,**h**) TEM images of the FCCF-2 sponge. (**i**) HRTEM image of the graphitized carbon layers on the alloy NPs’ surface. (**j**) SAED image corresponds to (**i**).

Figure 2a–c display the cross-sections of the FCCF-2 sponge with different magnifications, which are composed of multiple fibrous layers stacked internally. Numerous continuous filaments are intertwined and randomly stacked to form an ordered structure. The fibrous layers are connected by vertically arranged nanofibers. The internal short fibers may result from artificial cuts in the cross-section. Figure 2d–f and Figure S3 (Supporting Information) illustrate that the size and spacing of FeCoNi NPs can be tuned by changing the concentration of metal compounds in the spinning solution, which further affects the state of CNTs on the CNF surfaces.

The FCCF-1 sponge exhibits finer CNT with higher density, which may result from the lowest concentration of metal compounds (Figure 2d and Figure S3a). A higher concentration of metal compounds will gradually enlarge the FeCoNi alloy NPs and their number. As shown in Figure 2e and Figure S3b (Supporting Information), the growth mode of CNTs belongs to the tip growth mechanism, demonstrating that larger catalyst NPs could form thicker CNTs in FCCF-2 sponges. Hydrocarbon gas was cracked to produce carbon radicals on the surface of catalyst NPs at high temperatures. Then, carbon atoms precipitated on the catalyst NPs’ surface, forming hexagonal network graphite layer structures when the solubility of carbon radicals was exceeded. Noteworthy, the alloy catalytic system multi-metal alloy system has been demonstrated to enhance the high-temperature structural stability of catalyst NPs, inhibit Ostwald ripening of the NPs [40], and effectively regulate the binding energy of the catalyst to carbon [41], thereby enhancing the catalytic efficiency. Once the diameter of the catalyst NPs exceeds a critical threshold, their specific surface area and catalytic activity are diminished, making it impossible to catalyze the formation of CNT. As a result, the FCF sponge no longer exhibits the presence of CNT (Figure 2f and Figure S3c).

Furthermore, the composition of the FCCF sponge was characterized in detail. The elemental mapping in Figures S4 (SEM-EDX, Supporting Information) and S6 (HAADF-EDX, Supporting Information) illustrated that Fe, Co, and Ni were distributed evenly on the CNFs’ surface, further confirming the FeCoNi alloy NPs. Notably, the alloy elements were primarily concentrated at the tip of a single CNT by the tip growth of CNT. As Figure 2g–i and Figure S5 (Supporting Information) illustrate, alloy NPs are present at the tip of CNTs and on the surface of CNFs (FCCF-2 sponge). In the HRTEM image, lattice fringes of 0.202 nm and 0.354 nm can be observed, corresponding to the FeCo-based alloy (110) and graphitic carbon (002) planes, respectively [23]. It is visible that FeCo-based alloy NPs on the CNF’s surface are surrounded by a graphitized carbon layer, indicating that the magnetic alloy NPs can facilitate the conversion to sp²-hybridized carbon. Moreover, the selected area electron diffraction (SAED) image (Figure 2j) displayed polycrystalline rings of FeCo-based alloy, consistent with Figure 2i. Consequently, a multitude of heterogeneous interfaces (carbon/alloy particles, crystalline/amorphous interfaces) and lattice defects (e.g., point defects and dislocations) existed in the FCCF-2 sponge, which can influence the distribution of space charge and exacerbate polarization loss.

**Figure 3:**
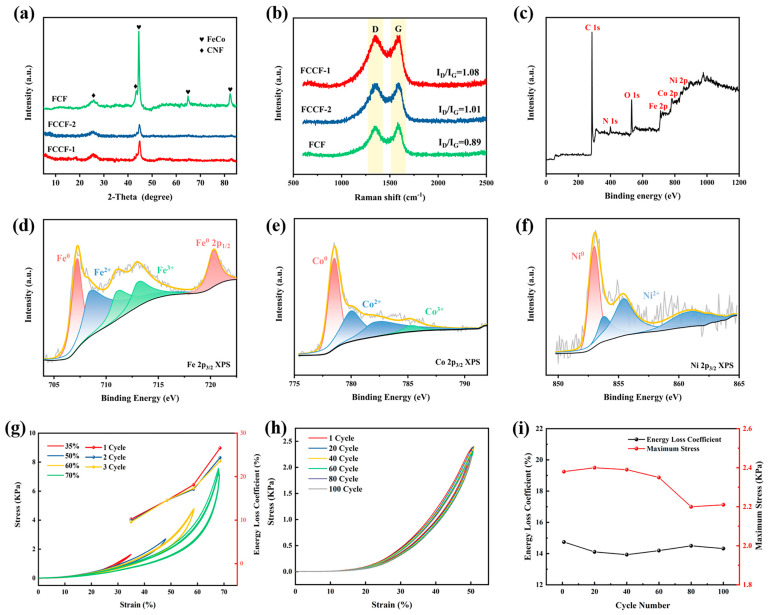
(**a**) XRD patterns of the FCCF sponges. (**b**) Raman spectra of the FCCF sponges. (**c**) XPS spectra of the FCCF-2. High-resolution XPS spectra of (**d**) Fe 2p_3/2_, (**e**) Co 2p_3/2_, and (**f**) Ni 2p_3/2_ for the FCCF-2. (**g**) Compression stress–strain curve and energy loss coefficient of the FCCF-2 sponge under 35, 50, 60, and 70% strain three times. (**h**) Compression stress–strain curve of the FCCF-2 sponge under 50% strain with a hundred cycles. (**i**) Energy loss coefficient and maximum stress of 50% compression strain with 100 cycles.

The XRD patterns of FCCF-1, FCCF-2, and FCF are displayed in Figure 3a. Notably, the prominent peaks observed at 25.8° and 42.4° corresponded to the (002) and (100) planes of graphitized carbon (PDF# 04-007-2081), resulting from the carbonization of PAN [42]. Furthermore, apparent peaks at 44.9°, 65.3°, and 82.7° correspond to the (110), (200), and (211) planes of the FeCo-type body-centered cubic structure (PDF# 00-049-1568) [23]. It is speculated that the Ni atoms may have entered the FeCo phase through displacement solution or interstitials. The peaks of the FeCo-based alloy for the FCF sponge are more precise and sharper, indicating higher crystallinity of the alloy NPs, which is attributed to the larger metal NPs’ size.

Figure 3b presents the Raman spectra. The curves exhibit enhanced resonance peaks at approximately 1350 cm^−1^ and 1580 cm^−1^, corresponding to the D and G peaks, respectively [43]. The D and G peaks reveal the lattice structure and chemical composition, where defects cause the D peak, while the G peak is mainly related to graphitic carbon [44]. The I_D_/I_G_ ratio typically quantifies the degree of graphitization. The I_D_/I_G_ values of the FCCF-1, FCCF-2, and FCF samples were 1.08, 1.01, and 0.89, respectively. As the quantity of metal increased, more alloys could engage in the graphitization transition, which follows the findings of the XRD pattern. Higher graphitized carbon will enhance conductivity and contribute to conductive loss. Furthermore, the defect sites contained in the CNF will act as polarization centers, which is crucial for improving polarization loss.

The XPS analysis was used to study the sample surface’ chemical composition and valence state. Figure 3c shows several typical peaks in the samples, such as Fe 2p, Co 2p, Ni 2p, C 1s, O 1s, and N 1s. In Figure 3d, the Fe 2p spectra are fitted to three Fe states; the peak with the binding energy of 707.2 eV and 720.3 eV belongs to the metallic Fe, the peak at 708.7 eV can be attributed to Fe^2+^, and the peaks at 711.3 eV and 713.3 eV correspond to Fe^3+^ [45,46]. Meanwhile, in the Co 2p spectra (Figure 3e), the peak at 778.5 eV is related to Co^0^, the peak at 780.1 eV and 782.7 eV are indexed to Co^2+^, while 785.4 eV is associated with Co^3+^ [45,47]. As shown in Figure 3f, Ni 2p can be decomposed into Ni^0^ and Ni^2+^. The peak at 852.98 eV is the metallic Ni. In addition, apparent peaks can be observed at about 853.7 eV, 855.4 eV, and 860.9 eV, which guarantees the presence of Ni^2+^ [45,48]. Specifically, the high-valence states of metallic elements resulted from the oxidation of FeCoNi NPs’ surface. The peak at 523.2 eV in the O 1s spectrum (Figure S7, Supporting Information) confirms the possible presence of metal carbonates and oxygen vacancies. This may be due to the oxidation of the crystalline carbon layer outside the metal NPs. Additionally, the presence of oxygen vacancies introduces polarization centers that contribute to the polarization loss.

Nitrogen adsorption and desorption experiments were carried out to characterize the textural properties of FCCF sponges. The isotherms depicted in Figure S8a (Supporting Information) exhibit characteristics of both type I and type IV mixtures [49,50], increasing rapidly at low pressure, slowing down with increasing pressure, and then rising rapidly again at high pressure [50]. Figure S8a,b (BJH analysis, Supporting Information) indicated the existence of both micro-pores and meso-pores within the FCCF-2 sponge. The formation of meso-pores may be attributed to the interstitial spaces between the CNF and CNT, whereas the micro-pores are associated with the pore structures of the CNF and are highly correlated with SEM and TEM results. Additionally, the FCCF-2 sponge with a hierarchical 3D structure exhibited a specific surface area of up to 148.463 m^2^/g and a total pore volume of 0.3045 cc/g, which is significant for multiple reflection and polarization loss.

### 3.2. High Elasticity of the FCCF Sponges

**Figure 4:**
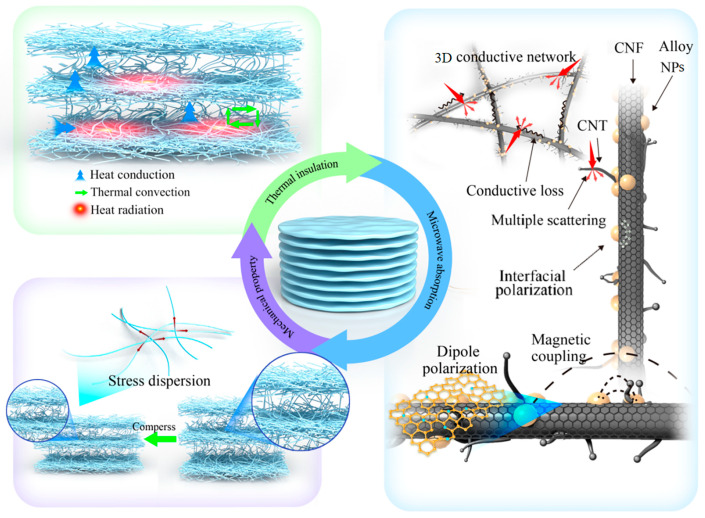
Schematic illustration of high elasticity, thermal insulation, and microwave absorption mechanisms for the FCCF sponges.

In practical applications of MA, structural stability and high elasticity are crucial [51]. Therefore, we evaluated the compressive behavior of the FCCF-2 sponge with a density of 9 mg·cm^−1^ at different strains and in cyclic compression at the same strain (Figure S9, Supporting Information). As illustrated in Figure 3g, the stress–strain curve of the sponge exhibits robust resilience properties during compression recovery. Even when compressed to 70% strain at room temperature, the FCCF-2 sponge exhibits a recovery of greater than 90%. The maximum compression stress of the FCCF-2 sponge at 35%, 50%, 60%, and 70% compression strain are 1.633 KPa, 2.712 KPa, 4.788 KPa, and 7.357 KPa, respectively, as shown in Figure 3g. After three compression cycles, the stress–strain curves exhibit a high overlap, with no discernible evidence of significant plastic deformation (Figure 3h). Figure 3g displays the energy loss coefficient for compression at various strain ratios ranging from 35% to 70%. When the compressive strain was increased from 35% to 70%, the energy loss coefficient changed from 9.5% to 26.5%, indicating that the FCCF sponge maintained structural integrity under significant strains. Figure 4 illustrates that compressive stresses are distributed throughout the three-dimensional stacked fiber layers, which enhances stress dispersion and allows the FCCF sponge to withstand more significant strains. Furthermore, the connected fibers exhibited a curling response to stress, whereby energy was stored and released as the stress was relieved, akin to a spring. These fibers cushion the stress at lower strain deformation, resulting in a low and stable energy loss factor. However, excessive strain damages the structure of fiber layers and connecting fibers, increasing the energy loss coefficient. We assessed the structural stability and fatigue resistance of FCCF sponges under strain cycling. Figure 3h illustrates that the structure retained its integrity after 100 cycles at 50% strain with slight plastic deformation. Damage to the connecting fibers was probably the cause of this deformation, yet the 3D structure remained stable. Figure 3i indicates that the energy loss coefficient remained at approximately 14% after 100 cycles. A notable decline of 0.15 KPa (6.4%) in maximum stress occurred at the 80th cycle, likely due to fiber deterioration. However, stress remained above 90%, demonstrating good fatigue resistance. The FCCF sponge exhibited better compression properties and fatigue resistance than existing materials [52,53].

### 3.3. Thermal Infrared Camouflage of the FCCF Sponges

**Figure 5:**
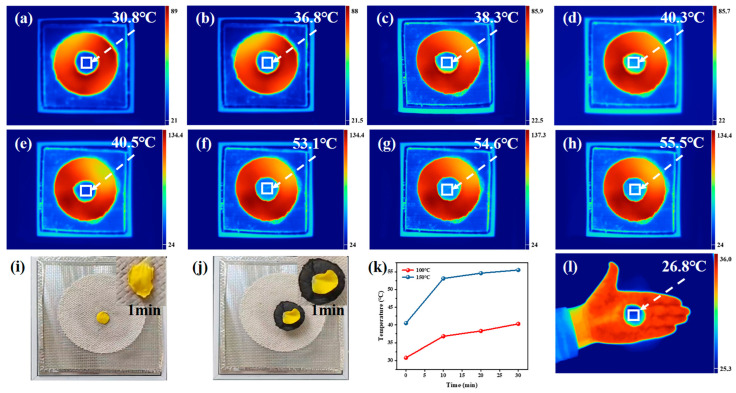
(**a**–**h**) Thermal infrared images of FCCF sponges on a heating platform captured at intervals of 10 min from 0 to 30 min. Yellow petals were placed onto the asbestos mesh directly (**i**) and the FCCF sponges (**j**), respectively. (**k**) Curve of sponge surface temperature as a function of time. (**l**) Thermal IR image illustrating the FCCF sponges used as infrared stealth materials.

The FCCF sponge, composed of FeCoNi alloy/CNT@CNF, can absorb and reflect infrared to achieve an infrared stealth effect, which is expected to achieve dual radar/infrared stealth. Furthermore, the as-prepared FCCF sponge contains multilayer fiber layers internally, with air still inside. The configuration yields robust thermal insulation performance, enabling the FCCF sponge to adapt to complex environments. As shown in Figure 5a–h, samples with a thickness of 3 mm were placed on a homemade heating table, and the temperatures were set to 100 and 150 °C, respectively. The surface temperature of the samples was observed using an infrared thermometer within 30 min of heating. The surface temperature of the samples increased slowly with heating time under different heating conditions. Figure 5k demonstrates a temperature difference of over 45 °C even after heating at 100 °C for 30 min and nearly 80 °C after heating at 150 °C for the same duration, indicating exceptional thermal insulation performance. Placing yellow petals on the heating table at 200 °C resulted in their curling up, hardening, and becoming brittle within a minute (Figure 5i). However, the petals placed on the 3 mm-thick sponge (Figure 5j) appeared unscathed, and the surface remained smooth and pliable as the multiple layers of stationary air isolated the heat. In contrast to conventional porous sponges, FCCF sponges contain a significant amount of stationary air between each fibrous layer. Figure 4 illustrates the potential heat transfer mechanisms of the prepared sponge. The three primary forms of heat dissipation in the sponge are heat conduction, heat radiation, and heat convection. (1) Heat conduction occurs in the solid phase or gas phase, which is believed to be the primary form of heat transfer within the sponge; (2) the thermal convection of gases is lessened due to the thinner air layer in each layer and the lower air content contained within the fiber layer, conducive to the thermal insulation effect; (3) thermal radiation from both hole walls and pores [54,55]. Furthermore, Figure 5k reveals that the sponge surface exhibits a dark blue coloration closely aligned with the surrounding environment when placed on the hand, suggesting that the sponge has powerful thermal infrared invisibility.

### 3.4. Microwave Absorption Performance of the FCCF Sponges

**Figure 6:**
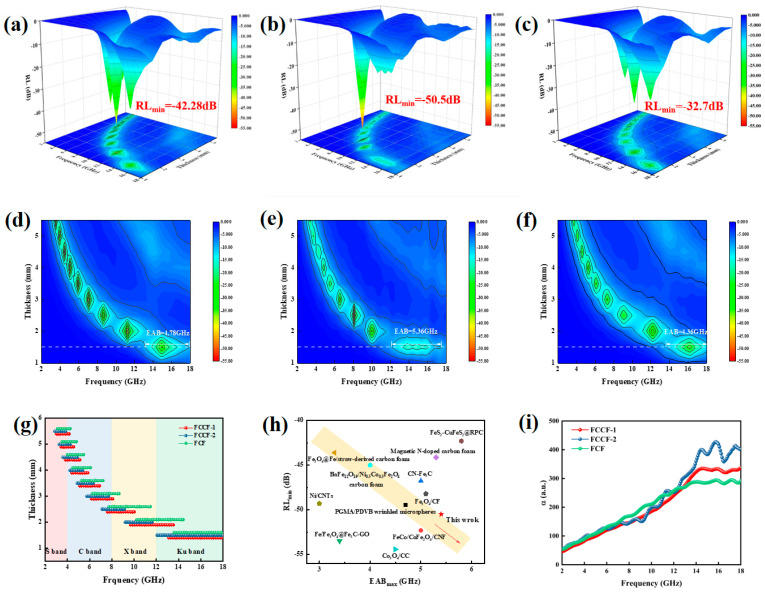
Reflection loss values images of (**a**) FCCF-1, (**b**) FCCF-2, and (**c**) FCF at 2–18 GHz. Two-dimensional representation of the reflection loss values of (**d**) FCCF-1, (**e**) FCCF-2, and (**f**) FCF at 2–18 GHz. (**g**) Effective absorption bandwidth at varying thickness for all samples. (**h**) Performance comparison of MA for different associated materials. (**i**) Attenuation constant α of the FCCF sponges.

According to transmission line theory, Equations (1) and (2) can calculate RL. As shown in Figure 6a–f and Figure S10a–c and Table 1, the FCCF-2 sample has an optimal RL of −50.5 dB at 8 GHz with a thickness of 2.5 mm, and its EAB_max_ can reach 5.36 GHz, which fully covers the entire Ku-band, demonstrating the material’s superior performance. Furthermore, Figure 6g presents a comparative plot of the effective absorption bandwidth at varying thicknesses for all samples, indicating that the FCCF-2 sample exhibits the most considerable absorption bandwidth among all samples. Additionally, all samples can achieve complete frequency band absorption from 2–18 GHz by adjusting thickness. Compared with previous MA materials based on carbon or metal (Figure 6h and Table 1) [56,57,58,59,60,61,62,63,64,65], FCCF sponges exhibit lower RL and larger EAB. The low filler loading of 30% and the relative thinness of the absorbers (about 1.5 mm) result in the FCCF sponge being more ideal and efficient in MA. The three-dimensional conductive network, consisting of CNTs grown in situ on the surface of carbon nanofibers, along with unique laminar structures present within the FCCF sponge, contributes to the improved microwave absorption properties. The enhancement is attributed to the establishment of a specialized magnetic coupling network facilitated by the modulation of alloy nanoparticles and the observed magneto-electric coupling behavior.

The microwave absorption mechanism can be explained by electromagnetic parameters, as illustrated in Figure S10 (Supporting Information). The MA properties are generally strongly influenced by its complex permittivity ($\varepsilon =\varepsilon^{\prime }+{j\varepsilon }^{\prime \prime }$) and complex permeability ($\mu =\mu^{\prime }+{j\mu }^{\prime \prime }$). Specifically, the ability to store EM energy can be characterized by the real part of the complex permittivity ($\varepsilon^{\prime }$) and the complex permeability ($\mu^{\prime }$). The imaginary parts of the complex permittivity ($\varepsilon^{\prime \prime }$) and complex permeability ($\mu^{\prime \prime }$) can be used to assess the ability to dissipate EM energy [66].

Figure S10a,b (Supporting Information) show the curves of complex permittivity versus frequency for all samples. The FCCF-2 samples contain smaller metal NPs evenly dispersed on the CNF’s surface, resulting in more carbon–metal interfaces and polarization centers. Additionally, the in situ-grown CNT lengthens the carrier conduction paths and contributes to more polarization centers [67]. The FCCF-2 sample produced a more efficient dielectric response mechanism, causing the real and imaginary parts of its complex permittivity to be the most prominent among all the samples. The average value of the real part of the complex dielectric constant of the FCCF-2 sample is 13.99, significantly higher than that of the FCCF-1 (11.70) and FCF (10.10) samples. Consequently, the FCCF-2 samples exhibit augmented electrical energy storage capacity, which may be attributed to the optimal alloy particle density and the CNT’s growth. The real part of the complex permittivity of all the samples shows a gradual decrease, which can be attributed to the dispersion effect [68]. Similarly, in the 2–18 GHz range, the FCCF-2 sample exhibits a higher imaginary part of complex permittivity, indicating a more excellent EMW loss capability than other samples. Additionally, all curves show more fluctuations in the X-band and Ku-band, suggesting abundant interfacial and dipole polarization in the sponge, which improves the material’s microwave absorption properties [69]. Furthermore, the dielectric loss angle tangent ($tan\delta_{\varepsilon }=\varepsilon^{\prime \prime }/\varepsilon^{\prime }$) exhibits similar resonance peaks, providing additional evidence of multiple polarization behaviors (Figure S10c, Supporting Information).

**Figure 7:**
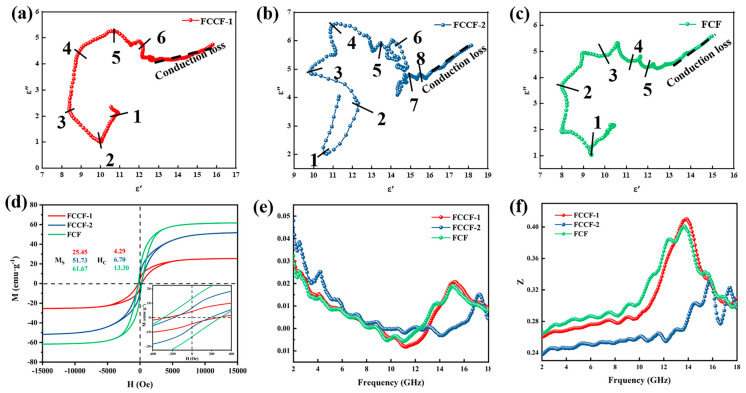
Cole–Cole plots of FCCF-1 (**a**), FCCF-2 (**b**), FCF (**c**). (**d**) Hysteresis loops of FCCF-1, FCCF-2, and FCF. (**e**) C_0_ values of FCCF-1, FCCF-2, and FCF. (**f**) Impedance-matching parameters of FCCF-1, FCCF-2, and FCF.

According to Debye’s relaxation theory, the real part and imaginary part of the complex permittivity can be expressed by Equations (3)–(5). The Cole–Cole semicircle was obtained by taking the real part and imaginary part of the dielectric constant as the x-axis and y-axis, respectively (Figure 7a–c). In general terms, the Cole–Cole curve is an irregular arc influenced by conductivity. The presence of a semicircle means that a polarization process is taking place [70]. There exist 6, 8, and 5 semicircles for FCCF-1, FCCF-2, and FCF samples, respectively, which suggests that the Debye relaxation behavior is more pronounced in FCCF-2, mainly owing to more complex interfaces and more defects. Conduction loss is also not negligible in the 3D interconnected network structure. The straight-line segment at the tail end of the Cole–Cole semicircle curve represents the conduction loss. As illustrated in Figure 7, all three samples exhibit extended dashed lines, which signifies that they possess robust conduction loss capability. Based on the observed polarization and conductive loss capacity, the FCCF-2 may exhibit the best dielectric loss capacity [42].

Figure S10d,e (Supporting Information) display the complex permeability of all samples. Each curve exhibits multiple resonance peaks in the low-frequency range and strong resonance peaks at approximately 14 GHz, corresponding to the natural resonance and exchange resonance, respectively [69]. The internal charges of the magnetic parts are irregularly moving due to the alternating EM field, causing a change in the internal energy of the composites. FCCF-2 demonstrates the highest magnetic loss tangent ($tan\delta_{\mu }=\mu^{\prime \prime }/\mu^{\prime }$), indicating the strongest magnetic loss performance. The $tan\delta_{\varepsilon }$ values are greater than those of $tan\delta_{\mu }$, revealing that the EMW loss mechanism in the FCCF sponge is predominantly dielectric loss [71]. Figure 7d shows the hysteresis loops of the sponges. The saturation magnetization (M_S_) values of the FCCF-1, FCCF-2, and FCF samples are 25.45, 51.73, and 61.67 emu·g^−1^, respectively. The coercivity (H_C_) values of the samples are 4.29, 6.70, and 13.30 Oe, respectively. As the size of alloy NPs increased, larger magnetic domain structures were formed more integrally, enhancing the *M_S_* and *H_S_* of the FCF sponge. The small-sized magnetic NPs create a unique magnetic vortex structure and couple with each other to form a dense magnetic coupling network, which may be why FCCF-1 and FCCF-2 also have intense magnetic loss [71]. As shown in Figure S11, all samples present high conductivity. The better conductivity gives FCCF sponges a high conductive loss, and the internal three-dimensional conductive network means they still have good microwave absorption performance at a low filler ratio. However, it also makes the impedance matching poor.

In the frequency range of 2–18 GHz, the main mechanisms of magnetic loss are eddy current loss, natural resonance, and exchange resonance. The eddy current coefficient C_0_ value variation represents the primary mechanism of magnetic loss (Equation (6)). Specifically, the C_0_ value decreases as the frequency increases, indicating that the natural resonance mechanism is predominantly involved. If the C_0_ value remains constant, it is predominantly associated with eddy current losses. Figure 7e illustrates that C_0_ exhibits a decreasing trend with frequency in the 2–18 GHz band, accompanied by considerable fluctuations. This indicates that the magnetic loss mechanisms of sponges are predominantly exchange resonance and natural resonance. Concurrently, the in situ reduction method ensures that the alloy NPs are distributed uniformly throughout the carbon fibers, thereby preventing the agglomeration of magnetic NPs. The wrapping of the graphitized carbon layer effectively prevents the generation of eddy currents, suppressing the skin effect and enhancing the magnetic response of the isolated magnetic NPs.

The attenuation constant α is a crucial parameter that describes a material’s ability to reduce EMW, calculated by Equation (7). Figure 6i illustrates the attenuation coefficients of all samples, and FCCF-2 exhibits the highest attenuation capacity. As shown in Figure 7f, the FCCF-1 and FCF sponges exhibit less microwave reflection. Although the FCCF-1 sponge exhibits greater CNT and magnetic coupling behavior, its MA performance remains inferior to that of the FCCF-2 sponge. Polarization loss is assumed to play a more significant role in the loss system. Similarly, although FCF exhibits a higher saturation magnetization intensity and approximately equivalent conductive loss compared to FCCF-2, CNTs occupy a significant position in attenuating EMW. In conclusion, the FCCF-2 sponge exhibits excellent MA performance, which can be attributed to its strong polarization loss, excellent conductive loss, and magnetic loss.

Figure 4 illustrates the potential MA mechanism of the FCCF sponge. Firstly, owing to the hierarchical 3D interconnected network, the sponge contains a significant amount of air, which optimizes its impedance-matching properties. Furthermore, 3D structures can induce multiple reflections, prolonging the path of EMW. Secondly, the FCCF sponge consists of numerous CNT @ CNF structures, effectively lowering the percolation threshold and inducing the micro-currents, conducive to conduction loss. Thirdly, CNF and CNT are dielectric nanomaterials, possessing defect sites due to quantum size effects, which can cause the electric dipoles to move with the electric field. As the electric field frequency increases, the dipole’s response lags behind and eventually reaches a limit, resulting in polarization relaxation loss. Fourthly, heterogeneous interfaces were formed between the CNF, magnetic alloy NPs, and CNT, which can influence the space charge distribution and induce interface polarization. Fifthly, adjacent FeCoNi NPs on the surface of the CNF can induce magnetic coupling to achieve a spatial magnetic network, producing an intense magnetic loss to attenuate EMW.

### 3.5. Radar Stealth Performance Evaluated by RCS Simulation

**Figure 8:**
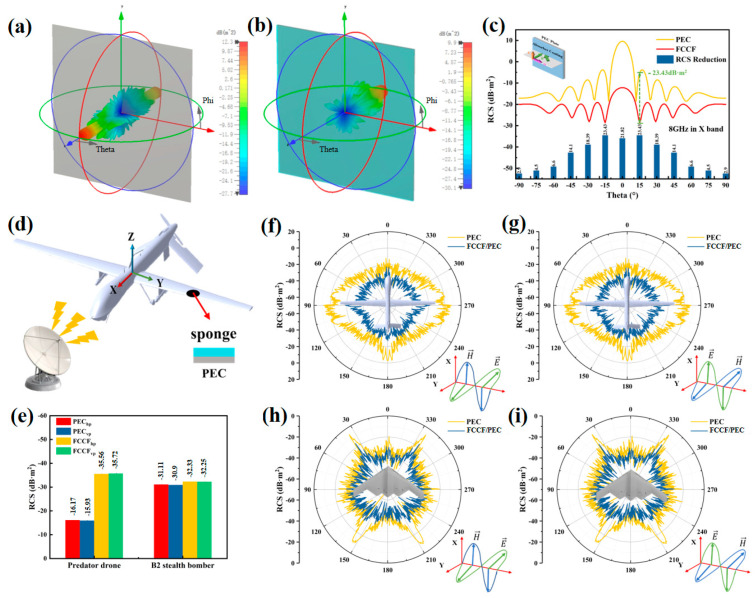
CST was used to simulate RCS (**a**) without and (**b**) with FCCF MA stealth protection. (**c**) RCS simulated curves of the PEC back plate and the FCCF sponge within different plane-wave angles (−90°~90°). (**d**) Schematic diagram of predator drones coated with the FCCF sponge. (**e**) Average RCS values of predator drone and B2 stealth fighter without and with FCCF coating at a frequency of 8 GHz. RCS radiation curves of predator drones coated with the FCCF sponge within different plane-wave angles at 8 GHz under horizontally polarized (**f**) and vertically polarized (**g**) waves. RCS radiation curves of B2 stealth fighter coated with the FCCF sponge within different plane-wave angles at 8 GHz under horizontally polarized (**h**) and vertically polarized (**i**) waves.

To assess the prospective radar stealth application for the designed FCCF MA sponges, we employed CST Studio Suite 2022 software to simulate the response to far-field EMWs. According to the simple simulation model, the incident EMW enters along the negative direction of the *Z*-axis and at an angle of theta for reception. Figure 8c illustrates that the FCCF markedly diminishes radar reflected signals within the −90° to 90° detection angle range at 8 GHz, thereby conferring radar stealth performance. Figure 8c displays that the FCCF has significantly alleviated radar reflection signals at detection angles ranging from −90° to 90° at 8 GHz, indicating that FCCF can effectively reduce the RCS value, thereby achieving radar stealth performance. As the detection angle varied, the RCS value declined gradually, presenting regular fluctuations. With an FCCF coating thickness of 2 mm, the RCS values decreased below −9 dB. Specifically, when the detection angle was 15°, the RCS attenuation value could reach a maximum of 23.43 dB·m^2^.

A predator drone model with 44.7 m in length and 28.3 m in width and a B2 stealth fighter model were established, as depicted in Figure 8d. The RCS values for the drone coated with the FCCF sponge are presented in Figure 8f,g, which show the RCS values within the plane-wave angles from 0° to 360° at 8 GHz. In general, the curve is closer to the center, suggesting more effective in stealth. The uncoated drone shows the most considerable RCS value, while a significant decrease is observed after applying the FCCF sponge. The average RCS values (Figure 8e) of the uncoated predator drone are −16.17 and −15.93 dB·m^2^, while that of the predator drone coated with FCCF are −35.56 and −35.72 dB·m^2^ under horizontal and vertical polarization, respectively. According to Equations (9) and (10), the radar detection distance of the FCCF MA stealth predator drone will be shortened to approximately 1/3 of the original. Compared with predator drones, the predator drone coated with FCCF exhibits a higher attenuation of more than 19 dB·m².

Owing to the unique shape, which gives the B2 stealth fighter a strong radar stealth capability, the decrease in RCS simulated values for the B2 stealth fighter with FCCF coating could have been more obvious. However, a similar RCS attenuation tendency was still achieved by coating the B2 Stealth Bomber with a 2 mm FCCF sponge (Figure 8e,h,i), indicating that the FCCF sponge has the potential to achieve the radar stealth effect in military fields.

## 4. Conclusions

In summary, self-supporting 3D architectures composed of FeCoNi alloy/CNT @ CNF were fabricated by a simple conjugated electrostatic spinning technique and then heat treatments, which exhibited an optimum RLmin value of −50.5 dB at 8 GHz and an EABmax of 5.36 GHz with 1.5 mm. The excellent MA performance originated for the following reasons: (1) the 3D hierarchical structure can induce multiple reflections, prolonging the path of EM waves; (2) the CNT @ CNF conductive network is conducive to conduction loss; (3) CNFs and CNTs are dielectric nanomaterials, resulting in polarization relaxation loss; (4) heterogeneous interfaces can influence the space charge distribution and induce the interface polarization; (5) adjacent FeCoNi NPs can induce magnetic coupling to achieve magnetic loss. Furthermore, the FCCF sponge possessed an attractive infrared stealth performance, ascribed to the following reasons: (1) CNTs can absorb and reflect infrared effectively, (2) the 3D porous structure, with abundant still air inside, has satisfactory thermal insulation performance. Therefore, the FCCF sponge simultaneously fulfills the requirements for microwave absorption and infrared stealth performance, indicating significant potential for applications in smart wearables, military technology, and other related area.

## Figures and Tables

**Table 1:** Comparison of the MA properties of various related materials.

Sample	Thickness (mm)	EAB (GHz)	RL (dB)	Reference
Fe_3_O_4_@Fe/straw-derived carbon foam	4.7	3.3	−43.6	[57]
Ni/CNTs	3.0	2.96	−49.3	[58]
CN-Fe_3_C	2.0	5.01	−46.78	[56]
FeS_2_-CuFeS_2_@RPC	1.9	5.78	−42.3	[59]
Fe/Fe_3_O_4_@Fe_3_C-GO	3.43	3.4	−53.5	[61]
Co_3_O_4_/CC	2.46	4.54	−54.43	[60]
PGMA/PDVB wrinkled microspheres	1.9	4.7	−49.45	[62]
Magnetic N-doped carbon foam	1.53	5.3	−44.15	[63]
BaFe_12_O_19_/Ni_0.5_Co_0.5_Fe_2_O_4_ carbon foam	2.0	4	−45	[64]
FeCo/CoFe_2_O_4_/CNF	1.95	5	−52.3	[65]
FCCF-1 sponge	2.0	4.78	−42.28	This work
FCCF-2 sponge	1.5	5.36	−50.5	This work
FCF sponge	2.0	4.36	−32.7	This work

## Data Availability

The original contributions presented in this study are included in the article and Supplementary Materials. Further inquiries can be directed to the corresponding author.

## Supplementary Materials

The following supporting information can be downloaded at: https://www.mdpi.com/article/10.3390/ma18010113/s1, Figure S1: (a) TGA curve and (b) DTG curve of the FCCF-2 sponge; Figure S2: A photograph of the lightweight FCCF-2 sponge which can stand on the stamen; Figure S3: SEM images of the (a) FCCF-1, (b) FCCF-2, (c) FCF; Figure S4: (a) SEM image of single fiber in the FCCF-2 along with energy dispersive X-ray (EDX) elemental mappings of (b) Fe, (c) Co and (d) Ni; Figure S5: TEM images of the FCCF-2 sponge; Figure S6: High-angle annular dark-field (HAADF) image of FCCF-2 sponge along with EDX elemental mappings of C, Fe, Co and Ni; Figure S7: High-resolution XPS spectra of O 1s for the FCCF-2 sponge; Figure S8: N_2_ absorption/desorption isotherms and the pore size distribution of the FCCF-2 sponge; Figure S9: The compression test for FCCF sponge, it rapidly returns to its original shape; Figure S10: (a) Reflection loss curves of FCCF-1, FCCF-2, and FCF sponge under different thicknesses of 1–5 mm. (d,e) Frequency-dependent value curves of real part of permittivity (ε′) and imaginary part of permittivity (ε″) of all samples. (f) The dielectric loss angle tangent of all products. (g,h) Frequency-dependent value curves of real part of permeability ($\mu^{\prime }$) and imaginary part of permeability ($\mu^{\prime \prime }$) of all products. (i) The magnetic loss tangent of all samples; Figure S11: The conductivity of FCCF-1, FCCF-2, and FCF.
