# Supplementary material for: Hierarchical 3D FeCoNi Alloy/CNT @ Carbon Nanofiber Sponges as High-Performance Microwave Absorbers with Infrared Camouflage
Source: Materials (Basel). 2024 Dec 30;18(1):113. doi: 10.3390/ma18010113 (PMC11720863; doi:10.3390/ma18010113)
Supplement: Supplementary file 1 [file materials-18-00113-s001.zip › materials-3359740-supplementary.pdf]

Supporting Information

Hierarchical 3D FeCoNi alloy/CNT @ carbon nanofiber sponges as high-performance microwave absorbers with infrared camouflage

Yifan Fei[1], Junya Yao[1], Wei Cheng[1], Wenling Jiao[1]*

[1] Shanghai Frontiers Science Research Center of Advanced Textiles, Engineering Research Center of Technical Textiles (Ministry of Education), Key Laboratory of Textile Science & Technology (Ministry of Education), College of Textiles, Donghua University, Shanghai 201620, China.

* Email: wenlingjiao@dhu.edu.cn

Supporting Figures S1-S11

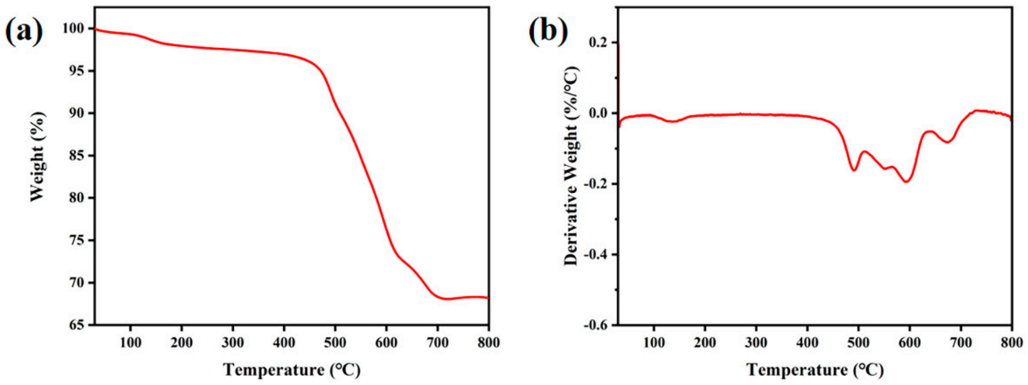

Figure S1 (a) TGA curve and (b) DTG curve of the FCCF-2 sponge.

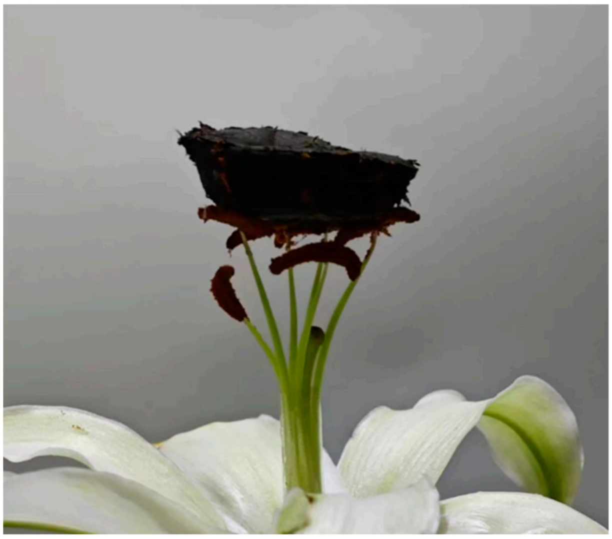

Figure S2 A photograph of the lightweight FCCF-2 sponge which can stand on the stamen.

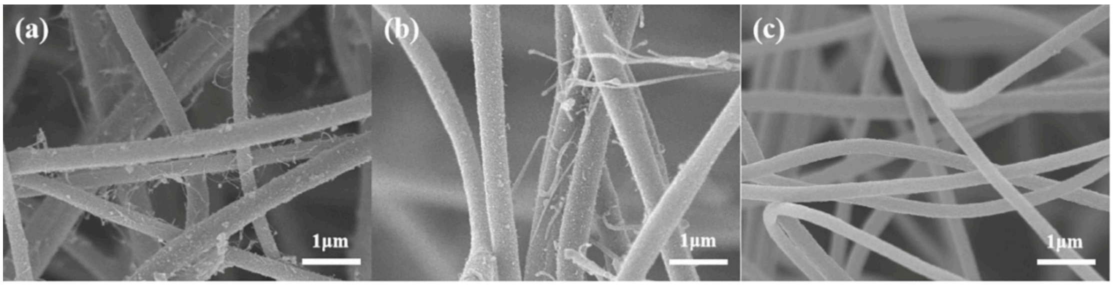

Figure S3 SEM images of the (a) FCCF-1, (b) FCCF-2, (c) FCF.

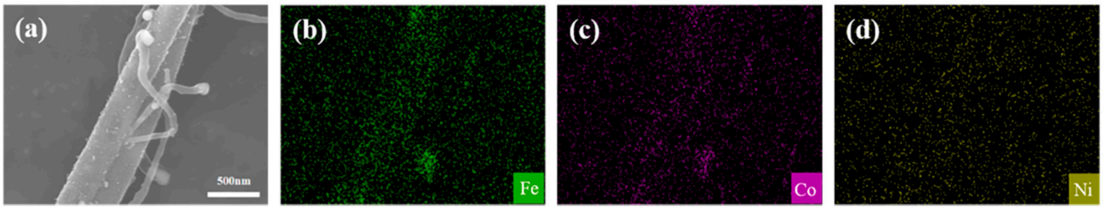

Figure S4 (a) SEM image of single fiber in the FCCF-2 along with energy dispersive X-ray (EDX) elemental mappings of (b) Fe, (c) Co and (d) Ni.

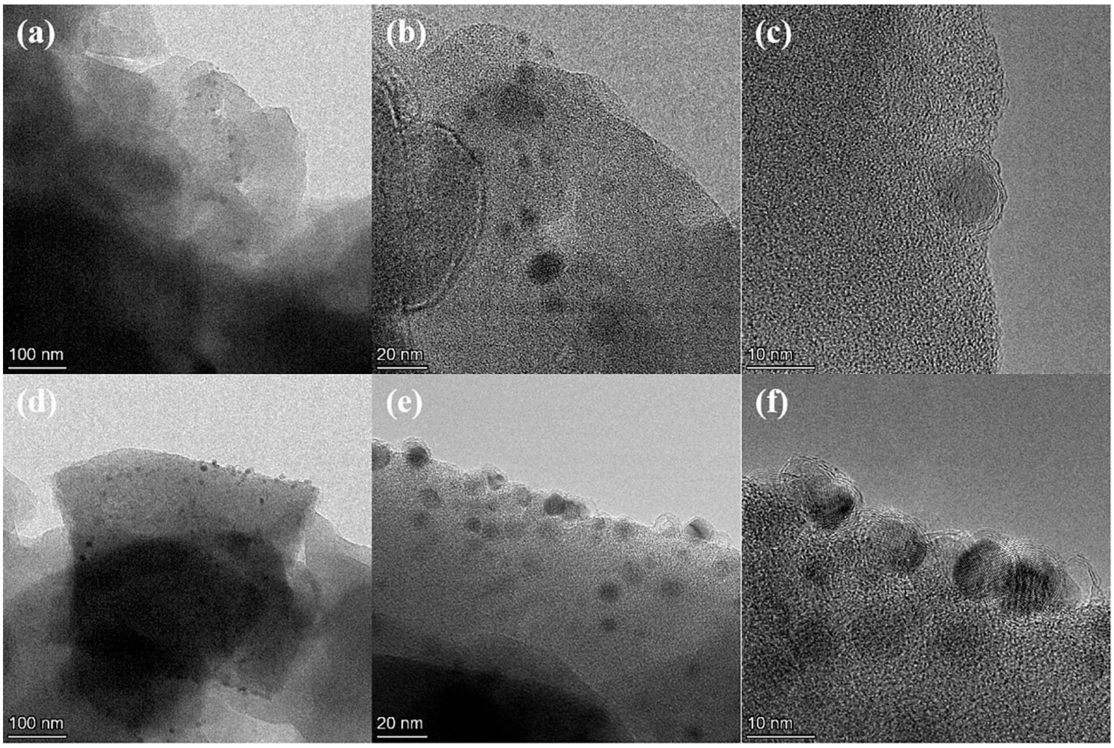

Figure S5 TEM images of the FCCF-2 sponge.

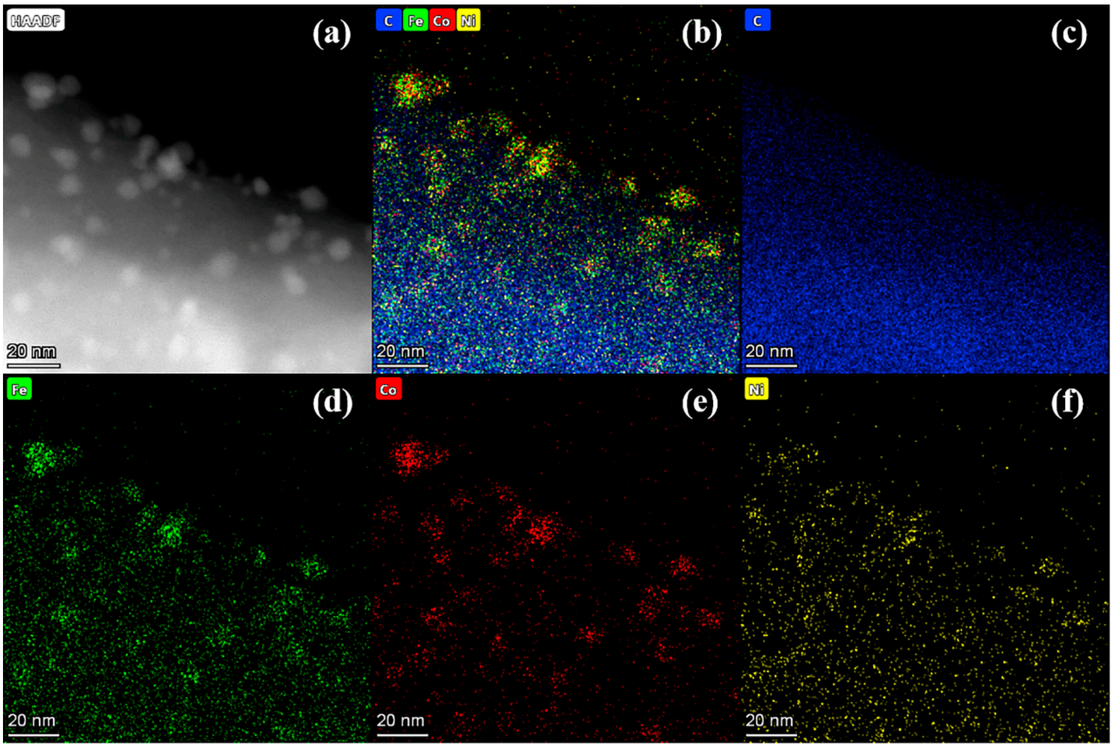

Figure S6 High-angle annular dark-field (HAADF) image of FCCF-2 sponge along with EDX elemental mappings of C, Fe, Co and Ni.

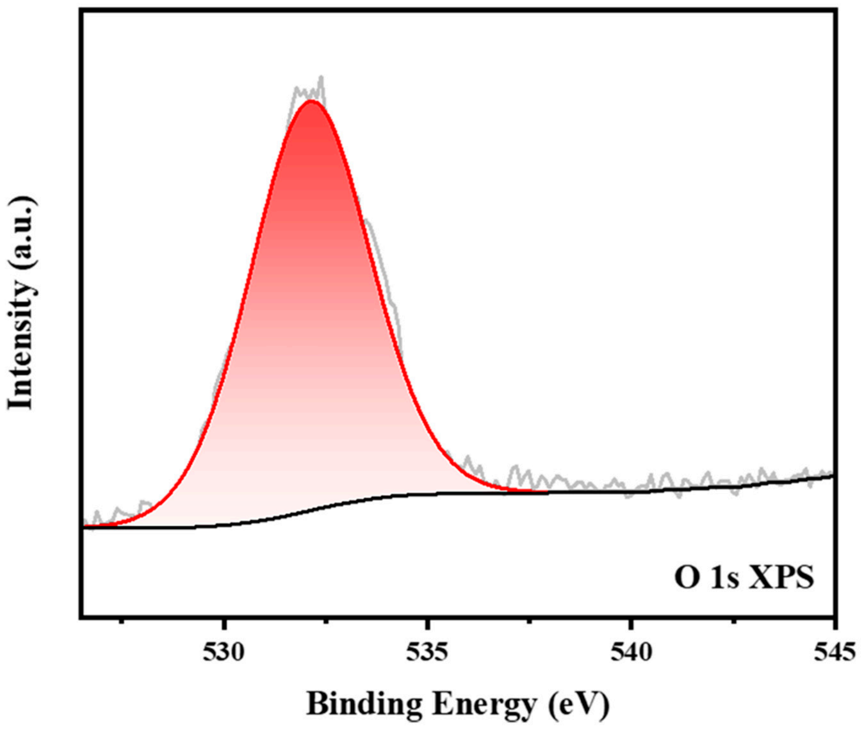

Figure S7 High-resolution XPS spectra of O 1s for the FCCF-2 sponge.

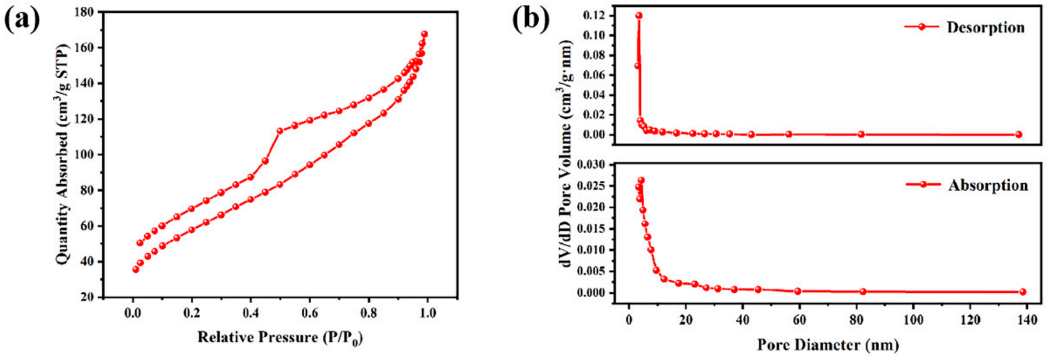

Figure S8 N_2 absorption/desorption isotherms and the pore size distribution of the FCCF-2 sponge.

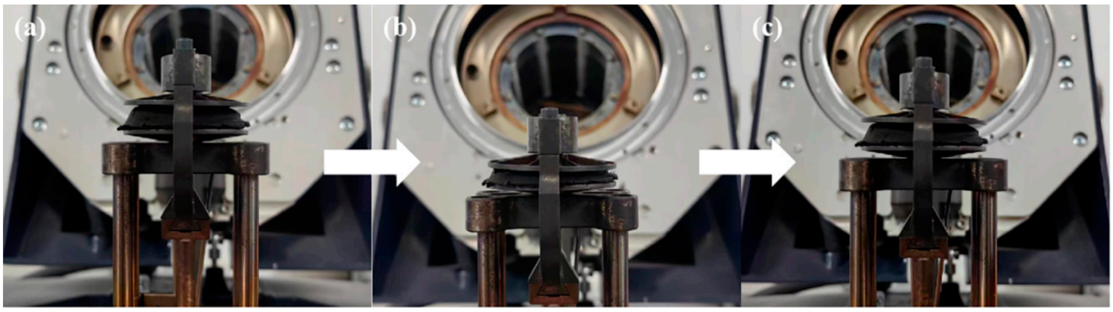

Figure S9 The compression test for FCCF sponge, it rapidly returns to its original shape.

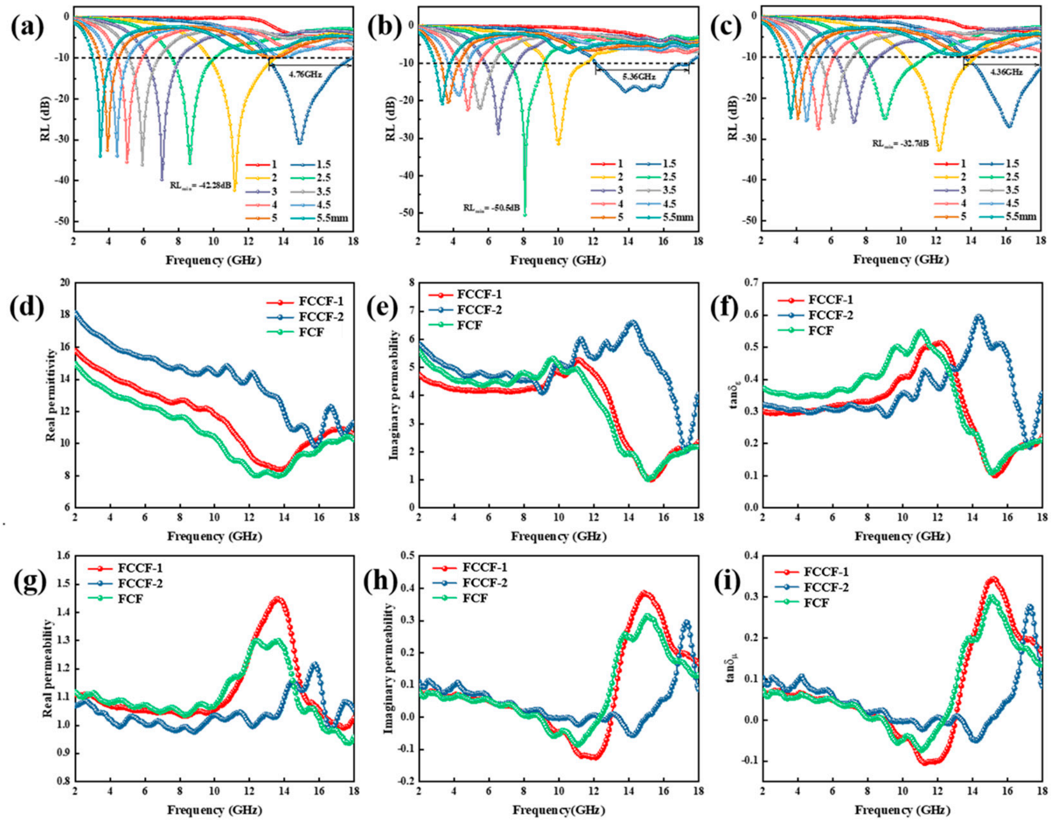

Figure S10 (a) Reflection loss curves of FCCF-1, FCCF-2, and FCF sponge under different thicknesses of 1–5 mm. (d), (e) Frequency-dependent value curves of real part of permittivity (ε′) and imaginary part of permittivity (ε″) of all samples. (f) The dielectric loss angle tangent of all products. (g), (h) Frequency-dependent value curves of real part of permeability (μ') and imaginary part of permeability (μ'') of all products. (i) The magnetic loss tangent of all samples.

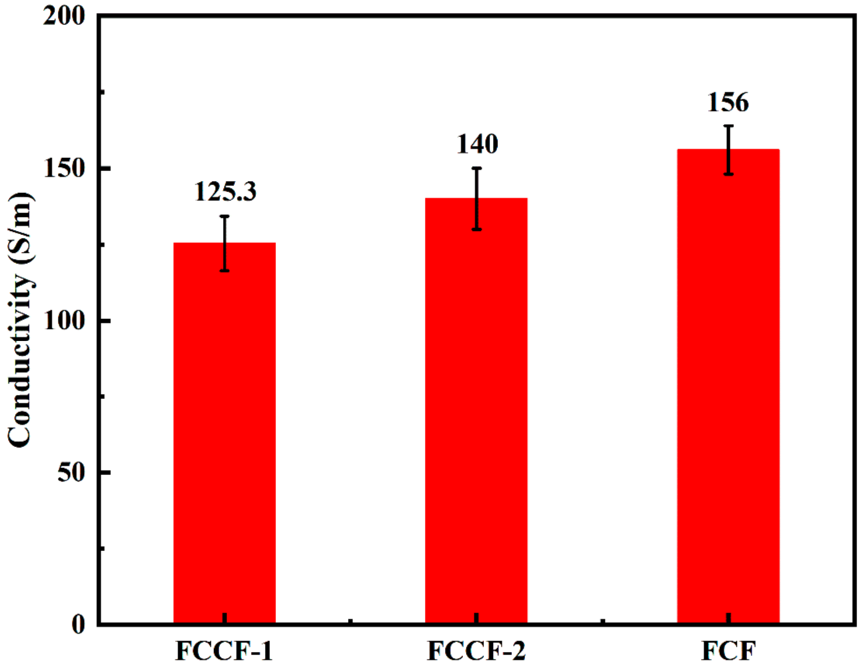

Figure S11 The conductivity of FCCF-1, FCCF-2, and FCF.
